# Dietary Total Antioxidant Capacity and Risk of Non-Alcoholic Fatty Liver Disease: A Case‐Control Study

**DOI:** 10.34172/jrhs.2020.18

**Published:** 2020-07-30

**Authors:** Ammar Salehi-Sahlabadi, Amin Mokari, Maryam Elhamkia, Fariba Farahmand, Masoumeh Jabbari, Azita Hekmatdoost

**Affiliations:** ^1^Student Research Committee, Department of Clinical Nutrition and Dietetics, Faculty of Nutrition and Food Technology, National Nutrition and Food Technology, Shahid Beheshti University of Medical Sciences, Tehran, Iran; ^2^Department of Community Nutrition, Faculty of Nutrition and Food Technology, Shahid Beheshti University of Medical Sciences, Tehran, Iran; ^3^School of Nutritional Sciences and Dietetics, Tehran University of Medical Sciences, Tehran, Iran.; ^4^Department of Clinical Nutrition and Dietetics, Faculty of Nutrition and Food Technology, National Nutrition and Food Technology, Research Institute, Shahid Beheshti University of Medical Sciences, Tehran, Iran

**Keywords:** Non-alcoholic fatty liver disease, Diet, Case-control studies

## Abstract

**Background:** Dietary total antioxidant capacity (DTAC) has been proposed as a tool for assessing the intake of antioxidants. This study aimed to assess whether a relationship exists between dietary total antioxidant capacity (TAC) and the odds of NAFLD.

**Study design:** A case-control study.

**Methods:** In this age‐and sex‐matched case‐control study in 2019, patients with NAFLD and healthy controls were recruited from a hospital clinic. All participants completed a validated 168‐ item food frequency questionnaire, the results of which were subsequently used to generate dietary TAC. Oxygen radical absorbance capacity values were used to calculate dietary TAC.

**Results:** Altogether, 225 patients with NAFLD and 450 healthy controls were enrolled. Participants with NAFLD had a higher mean weight, BMI, energy (*P* <0.050), and lower physical activity and DTAC scores (*P* <0.050) than the control group. In an adjusted model, participants who were in the highest quartile of dietary TAC had a lower risk of NAFLD (odds ratio 0.78, 95% CI: 0.67, 0.91).

**Conclusion:** A high DTAC was related to a decreased risk of NAFLD. Suggest the intake of a diet with high antioxidant capacity is significant at preventing NAFLD. Increasingly itemized investigations in design of randomized control trials require to reveal more insight into these results.

## Introduction

Non-alcoholic fatty liver disease (NAFLD) is the most popular type of chronic liver issue described by inordinate accumulation of fat in liver, and can advance to nonalcoholic steatohepatitis (NASH), fibrosis, cirrhosis, and hepatocellular carcinoma ^1^. The pervasiveness of NAFLD is expanding around the world. Roughly one billion people might be afflicted with NAFLD ^2^. The financial and healthcare burden of NAFLD was researched in an ongoing research. The creators coordinated 108,420 grown-ups with a first case for NAFLD to non-NAFLD controls. The whole yearly expense of care per another analyzed NAFLD persistent was $5506 higher than a coordinated control with the same metabolic comorbidities however without NAFLD ^3^. The expanding pervasiveness of NAFLD is an autonomous risk factor for type 2 diabetes, cardiovascular disease, chronic kidney disease, cirrhosis, liver cancer, and all-cause mortality^4^.

The pathophysiology of NAFLD is complicated and not found, however, is identified with undesirable ways of life, like physical inactivity and unhealthy diet ^5^. Grown-ups Diet is viewed as a significant factor in the improvement of NAFLD which various researches demonstrated that dietary vegetables and fruit utilization are conversely connected with NAFLD ^6,7^. Furthermore, a few researches have explored the significance of antioxidant intake to protect against oxidative harm and related inflammatory complications in NAFLD patients. Hence, high dosages of special antioxidants, for example, vitamins E, C and selenium are the topic of clinical trials^8,9^. Considering the focus of single antioxidants may not reflect the whole antioxidant power of food, the idea of total antioxidant capacity (TAC) was presented ^10^.

The TAC of foods indicates the cumulative capability of the whole dietary antioxidants agent to rummage free radicals, recommended as an instrument to explore the health impacts of antioxidants present in mixed diets ^11^. Dietary TAC has demonstrated advantageous impacts for degenerative disease, for example, cardiovascular disease, type 2 diabetes and cancer ^12-14^. Hence, there is deficient proof about the relationship between dietary TAC and danger of NAFLD. Thus, the purpose of the present investigation was to survey likely relationships among dietary TAC and NAFLD risk.

## Methods

### Study design and population

This case-control research was directed among patients who referred to a referral hospital in Isfahan (Al-Zahra Hospital in 2019). Subsequently, 225 patients influenced by NAFLD who referred to a hospital gastroenterology outpatient clinic and 450 non-NAFLD members who referred to hospital orthopedic and Ophthalmology centers were selected. Exclusion criteria were history of diseases such as renal and hepatic (Wilson's disease, autoimmune liver disease, virus infection, and alcoholic fatty liver), cardiovascular disease, malignancy, thyroid disorder, autoimmune diseases, and special dietary or physical activity regimens. Moreover, participants who did not complete more than 35 items of the food frequency questionnaire and those who reported total daily energy intakes ≤800 or ≥4500 kcal/d were selected.

All participants prepared informed consent preceding the investigation enlistment. A gastroenterologist following the laboratory tests and liver steatosis in the ultrasound analyzed the NAFLD. The control group individuals were coordinated with the patients in terms of their age and sex. Moreover, all patients experienced an ultrasound assessment and no proof of hepatic steatosis was seen among the control group. In this study, 225 people for case group and 450 for control group were selected. The study was reviewed and approved by the ethics committee (No: IR.SBMU.RETECH.REC.1398.10561) of Shahid Beheshti University of Medical Sciences and all participants provided written informed consent.

**Figure:**
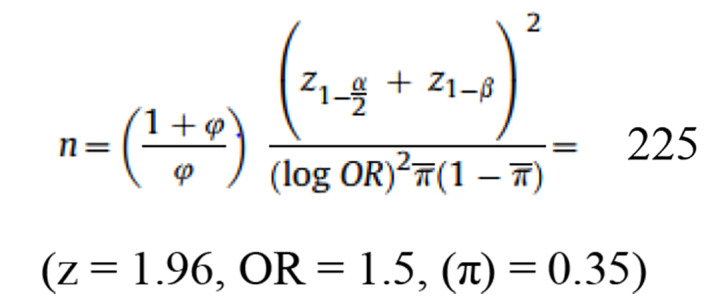


### Dietary assessment

Data on the diet of members was gathered utilizing a semi-quantitative food frequency questionnaire (FFQ) containing 168 food items ^15^. FFQ incorporated a list of typical Iranian foods with standard serving sizes. In this FFQ, the frequencies of devoured nourishments were changed over into every daily consumption. DTAC was determined dependent on the oxygen radical absorbance limit of every food, which information was then characterized to as µmol of Trolox equivalents/100 g of food (µmol TE/100 g) ^16^.

### Assessment of other variables

Weight was evaluated in standing, wore least clothes and without shoes. Their height was evaluated utilizing a mounted tape. The participant was again in a standing, wore least clothes and without shoes. Physical action was evaluated by IPAQ questionnaire (international action questionnaire) which information were then changed over to Metabolic Equivalent minutes out of every week (MET, min/week) ^17^. A prepared dietitian directed all the estimations to lessen mistake.

### Statistical analysis

All the statistical investigations were handled utilizing SPSS version 23 (IBM, Armonk, NY, USA). Continuous variables were noted as mean ± standard deviation that investigated utilizing χ^2^ test, though straight out factors were communicated as numbers and rates that broke down utilizing by autonomous examples t-test. Mean dietary admissions of members were thought about across quartile classes of DTAC utilizing the general direct model with change for age and vitality admissions. The odds ratio (OR) and 95% confidence interval (CI) of NAFLD in every quartile of total DTAC was characterized by conditional logistic regression models with adjustment for potential confounding variables in various models. Firstly, we didn't adjust for any potential confounders (model 1). Second we controlled for age (constant), sex (categorical) and education (under diploma, diploma, and bachelor, higher than bachelor) (model 2). At last, further adjustment was made for weight, WHR, BMI, physical action (MET-min/wk), marital status, fiber, vitamin C, vitamin D, zinc, energy (kcal/d) (model 3).

## Results

The features of control and NAFLD participants and dietary consumption are shown in Tables 1 and 2. Participants by NAFLD had a more mean weight, BMI, energy (*P* <0.050), and lower physical action and DTAC scores (*P* <0.050) compared to the control group (Table 1). Moreover, there was an important distinction in the intake of a handful of nutrients between two groups (Table 2). The meaningful difference was found in daily consumption of fiber, vitamin C, vitamin D and zinc among cases and controls so that cases had higher utilization of fiber (*P* =0.020) vitamin C, zinc (*P* =0.001) and took a lower vitamin D (*P* =0.010) than controls. The participants' features over the quartiles of DTAC are shown in Table 3.

**Table 1:** General characteristics of cases and controls participating in the study

**Continuous variables**	**Control group**		**Case group**		***P*** **value**
	**Mean**	**SD**	**Mean**	**SD**	
Age (yr)	37.88	8.92	38.63	8.71	0.720
Body mass index (kg/m^2^)	24.99	3.09	30.56	4.02	0.000
Waste hip ratio	0.87	0.06	0.97	0.06	0.480
Dietary total antioxidant capacity (mmol TE)	19491.58	9431.84	19536.28	462.31	0.660
Physical activity (MET-min/wk )	1590.30	949.44	1119.03	616.35	0.010
Energy (kcal)	2170.57	645.32	2315.46	621.24	0.004
**Categorical variables**	**Number**	**Percent**	**Number**	**Percent**	***P*** **value**
Marital status					0.001
Single	70	15.6	18	8.0	
Married	366	81.3	199	88.4	
Divorced/ widowed	14	3.1	8	3.6	
Education					0.001
Lower than diploma	49	10.9	33	14.7	
Diploma	186	41.3	91	40.4	
BSc	143	31.8	94	41.8	
Higher than BSc	72	16.0	7	3.1	
Smoking status					0.620
Nonsmoker	438	97.3	209	92.9	
Smoker	12	2.7	16	7.1	
Sex					0.970
Male	233	51.8	125	55.6	
Female	217	48.2	100	44.4	

**Table 2:** Daily macro- and micronutrients intakes in case and control group

**Variables**	**Control group**		**Case group**		***P*** **-value**
	**Mean**	**SD**	**Mean**	**SD**	
Carbohydrate (g)	311.25	99.04	331.68	99.47	0.711
protein(g)	73.49	23.87	78.22	24.01	0.702
Fat (g)	76.52	28.33	81.06	29.37	0.724
Fiber (g)	35.45	18.17	39.85	23.07	0.010
Cholesterol (mg)	225.48	141.62	223.41	116.78	0.662
Saturated fat (mg)	25.95	10.39	26.91	10.86	0.387
Monounsaturated fatty acid (mg)	26.41	10.24	28.29	10.83	0.702
Vitamin A. REA (mg)	485.18	304.32	458.65	316.40	0.950
Vitamin C (g)	135.68	85.60	139.12	83.91	0.001
Vitamin D (µg)	2.05	1.82	1.69	1.30	0.010
Folic acid(µg)	504.67	155.16	543.91	174.21	0.881
Vitamin 12 (mg)	3.94	1.12	4.07	2.39	0.192
Vitamin K (mg)	183.81	139.60	181.35	123.47	0.090
Zinc (mg)	10.77	3.64	11.53	3.61	0.001
Carbohydrate (g)	311.25	99.04	331.68	99.47	0.711
Protein(g)	73.49	23.87	78.22	24.01	0.702

**Table 3:** Characteristics of participants across quartiles of dietary total antioxidant capacity (µmol TE/100 g)

**Continuous variable**s	**Q1** **<12966.46**		**Q2** **12966.46-18010.15**		**Q3** **18010.15-23700.15**		**Q4** **>23700.15**		***P*** **trend**
	**Mean**	**SD**	**Mean**	**SD**	**Mean**	**SD**	**Mean**	**SD**	
Age (yr)	36.16	8.41	37.76	8.31	38.51	8.70	40.07	9.61	0.001
Weight (kg)	70.08	13.17	72.11	11.46	71.87	13.56	77.96	12.28	0.411
Body mass index (kg/m^2^)	26.41	4.19	27.01	3.81	27.10	4.62	26.86	4.59	0.472
Physical activity (MET)	1474.14	905.14	1371.69	783.86	1427.36	932.61	1451.80	899.32	0.730
Energy intake (kcal/d)	1824.73	541.23	2121.07	509.10	2377.99	609.11	2554.24	545.42	0.001
**Categorical variables**	**Number**	**Percent**	**Number**	**Percent**	**Number**	**Percent**	**Number**	**Percent**	***P*** **trend**
Sex									0.244
Male	89	13.2	92	13.6	79	11.7	97	15.7	
Female	79	11.7	76	11.3	89	13.2	71	14.4	

MET, metabolic equivalent task

There was no distinction in weight, BMI and physical action among DTAC quartiles. Across expanding DTAC quartiles, all participants had higher age and Energy admission (*P* <0.050). Correlations among DTAC and food groups involving fruits, vegetables, nuts, legumes, fruit juice, tea, and olive oil are introduced in Table 4. Furthermore fruit (r=0.75), vegetables (r=0.60), nuts (r=0.43), legumes (r=0.35), fruit juice (r=0.31), tea (r=0.29), and olive oil (r=0.16). Additionally, food groups, for example, vegetables (31.8%), fruits (26.4%), tea (11.8%), legumes (8.4%), fruit juice (2.1) and nuts (8.2%) were seen as the primary supporters of DTAC (Table 5). Multivariable-adjusted ORs and 95% CIs for NAFLD per every unit increment in dietary TAC just as across quartiles of dietary TAC scores are presented in Table 6. Contrasted and members in the most minimal quartile of DTAC, those in the most elevated quartile had a fundamentally lower OR for NAFLD (model 1: OR, 0.62; 95% CI: 0.51, 0.74). After adjustment for age, sex and education the results revealed that high DTAC score was a protective factor against NAFLD and participants with highest quartile of DTAC had 22% lower odds of NAFLD than those with lowest quartile (model 2: OR, 0.78; 95% CI: 0.67, 0.91).

**Table 4:** Correlation between dietary total antioxidant capacity and total antioxidant capacity from food groups

**Food groups**	**Partial correlations (r)**
Fruits	0.752
Vegetables	0.601
Nuts	0.432
Legumes	0.351
Fruit juice	0.310
Tea	0.290
Olive oil	0.164

**Table 5:** Contribution of food groups to overall dietary total antioxidant capacity (DTAC) intake (%)

**Nutrition item**	**%DTAC intake**
Fruit	26.4
Vegetables	31.8
Legumes	8.4
Fruit juice	2.1
Nut and seed	8.2
Tea	11.8
Olive oil	0.1

**Table 6:** Crude and Multivariable-adjusted odds ratios and their 95% confidence interval of the associations between Nonalcoholic fatty liver disease and dietary total antioxidant capacity

**DTAC (µmol TE)**	**Q1** **<15974.99**	**Q2** **15974.99 to 19815.70**	**Q3** **19815.70 to 25036.80**	**Q4** **>25036.80**	***P*** **trend**
Model 1	1	0.79 (0.65, 0.98)	0.68 (0.53, 0.82)	0.62 (0.51, 0.74)	0.033
Model 2	1	0.89 (0.75, 1.03)	0.84 (0.75, 0.94)	0.78 (0.67, 0.91)	0.041
Model 3	1	0.83 (0.73, 0.94)	0.81 (0.72, 0.92)	0.75 (0.64, 0.86)	0.043

Model 1: Crude. Model 2: Adjusted for age, sex, education. Model 3: Adjusted for age, sex, education, weight, waste hip ratio, body mass index, physical activity, marital status, fiber, vitamin C, vitamin D, zinc, energy.

Furthermore, after further adjustment for age, sex, education, weight, WHR, BMI, physical action, marital status, fiber, vitamin C, vitamin D, zinc and energy, compared to participants in the most minimal quartile of DTAC, and in the most elevated quartile had a meaningfully lower OR for NAFLD (model 3: OR, 0.75; 95% CI: 0.64, 0.86).

## Discussion

As far as we could know, the current research is one of the few researchers to consider the relation between DTAC and NAFLD for a case-control manner. In the current research, correlation of the dietary segments between NAFLD group and healthy controls demonstrated that there was meaningful contrast in daily consumption of fiber, vitamin C, vitamin D and zinc between two investigation groups. Likewise, there was higher utilization of fiber, vitamin C, zinc and lower utilization of vitamin D in NAFLD group than controls. Besides, food groups, for example, vegetables, fruits, tea, legumes, fruit juice and nuts were seen as the major supporters of DTAC in total investigation populace respectively. In the pathophysiology of NAFLD the role of oxidative damage has been established in previous researches^18,19^. In the liver damage, reactive oxygen species (ROS) are responsible for mitochondrial damage, reduced ATP, increment in apoptosis and peroxidation of lipids. In this process ROS are important by-product in oxidation of microsomes and peroxisomes ^20^.

Dietary components have important role in the oxidative and anti-oxidative situation of the body in the various diseases^21,22^. Moreover, these components have effective role in attenuation of some chronic diseases' complications^23,24^.

One hypothesis on these protective effects of dietary components is that all different types of antioxidants and their derivatives can effectively defense cells against ROS induced oxidative stress ^25^. In this regard, there are numerous dietary antioxidant components such as vitamin C, E and various phytochemicals^26,27^. Previous studies have proposed some beneficial role of DTAC in the function of lung ^28^ and endothelium ^29^. DTAC has inverse association with risk of some chronic diseases such as metabolic syndrome, rectal cancer and ischemic stroke and some cardiovascular disease (CVD) biomarkers ^25^.

Dietary antioxidants, especially when to originate from fruits and vegetables, were directly related to decreased risk of chronic diseases^30,31^.

In the present study, DTAC was an effective dietary predictor of intake of food groups with more antioxidant components such as vegetables, fruits, tea, legumes, fruit juice and nuts compared to other ones. On the other hand, DTAC had strong correlation with intake of fruits and vegetables. Nutritional recommendations for fruits and vegetable intake may have beneficial effects on NAFLD prevention and development of liver damage and steatosis.

Considering immediate and some strong correlation between DTAC and food categories involving fruits, vegetables, legumes and tea, the results of different researches are in understanding to these outcomes. High antioxidant content foods, for example, fruits, vegetables, legumes, tea and coffee are progressively connected with DTAC ^32^. DTAC has a significant direct relationship with the consumption of some dietary components and micronutrients involving fiber, folic acid, vitamins A, C, and E, total carotenoids, magnesium, and zinc^33,34^. Defected mechanisms of antioxidant defense can finally cause elevation in lipid peroxidation, cellular and enzyme damages and development of NAFLD^18,35,36^. Therefore, consumption of food groups specifically with higher content of mentioned components – such as fruits, vegetables, legumes and tea - might be involved in liver damage and NAFLD pathogenesis and prevention.

Another result of the current research was about DTAC in NAFLD patients. Subjects with NAFLD had a lower DTAC contrasted with the benchmark group. Subjects in higher DTAC quartiles had lower OR for NAFLD contrasted with the lower quartiles and being in most elevated DTAC quartiles had a defensive impact against NAFLD in crude and adjusted model. DTAC might be a likely indicator of the hazard for improvement of essential liver harms and activity oxidative circumstance in NAFLD. Additionally, lower consumption of some food groups involving fruits, vegetables and legumes in pre-diabetic members were related with oxidative circumstance ^25,32^. The results of the current investigation may feature the significance of DTAC in NAFLD and propose that dietary cell reinforcement substance might be answerable for anticipation of oxidative harm and movement of liver harms.

Having large sample size is worth noting strength of the present study. Although the present study is, one of the few case-control studies to evaluate the correlation between DTAC and risk of NAFLD but had some limitations including 1) as the present study had cross-sectional entity so it could not suggest any casual association between DTAC and NAFLD. 2) Using FFQ for dietary assessment in research studies exposed the estimation of food intake and quantification of the portion sizes to recall bias. 3) Antioxidant content of food items could be affected by different conditions from production to storage, processing and cooking conditions which could not assess by this type of study methods. 4) Like other case-control and cross-sectional studies, generalization of the findings of the current research to other samples and populations maybe not possible and applicable.

## Conclusion

A high DTAC was related to a decreased risk of NAFLD. The intake of a diet with high antioxidant capacity is significant at preventing NAFLD. Increasingly itemized investigations in design of randomized control trials require to reveal more insight into these results.

## Acknowledgements

This study is related to project NO. 1398/10561 From Student Research Committee, Shahid Beheshti University of Medical Sciences (SBMU), Tehran, Iran. We also appreciate the Student Research Committee and Research & Technology Chancellor in SBMU for their financial support of this study.

## Conflict of interest

The authors have no conflict of interest to declare.

## Funding

The authors sincerely thank Shahid Beheshti University of Medical Sciences for all moral and material supports. This study was supported by grants from the Student Research Committee Shahid Beheshti University of Medical Sciences (SBMU), Tehran, Iran (Grants ID: 1398/10561).

## Highlights

- High DTAC was associated with a reduced risk of non-alcoholic fatty liver disease in adults.
- Food groups such as vegetables, fruits, tea, legumes, fruit juice and nuts were found to be the main contributors to non-alcoholic fatty liver disease.
- Suggest of the intake of a diet with high antioxidant capacity is significant at preventing NAFLD.
